# Cohort profile of the first 2,000 canine enrolees in the Mars Petcare Biobank: demographic, hematologic and serum biochemistry results from March 2022 to December 2024

**DOI:** 10.1186/s12917-026-05419-6

**Published:** 2026-03-20

**Authors:** Janet E. Alexander, Charlotte Appleton, Sarah S. K. Beatty, Dottie C. Brown, Laura Carvell-Miller, Talon S. McKee, JoAnn Morrison, Janet C. Patterson-Kane, Rhiannon Reynolds, Susan Wadulack, The Mars Petcare Biobank Project Team, Katherine Bowen, Elisabete Capitao, Sara Cook, Mara DePena, Luisa De Risio, John Flanagan, Brenda Fulcher, Kristi Grace, Negin Habibi, Meredith Houk, Jodie Jackson, Amber Lynch, Sarah Moore, Corley-Anne Parker, Chad Rumsey, Stacy Smith, Lauren Wolfe

**Affiliations:** 1 Waltham Petcare Science Institute, Mars Petcare, Waltham On the Wolds, Melton Mowbray, Leicestershire, LE14 4RT UK; 2 Antech Diagnostics, Fountain Valley, CA USA; 3 Mars Veterinary Health, 18101 SE 6th Way, Vancouver, WA 98683 USA; 4 BluePearl Science, 2950 Busch Lake Blvd, Tampa, FL USA

**Keywords:** Dog, Cohort, Longitudinal, Health, Biochemistry, Haematology, Demographics

## Abstract

**Background:**

The MARS PETCARE BIOBANK™ (MPB) is a study recruiting pets visiting Mars Veterinary Health hospitals in the USA over a ten-year period, with the aim of analysing longitudinal data from thousands of otherwise healthy dogs and cats at their first presentation to identify novel and actionable pet health insights​. The present study summarises the baseline demographic, haematologic, and serum biochemistry data recorded for the first 2000 dogs enroled in the MPB study between March 2022 and December 2024 and considers how representative they are of the general population in the United States.

**Results:**

The median enrolment age was 3.0 years (0.5–10.0 yrs). The population was 52% male and 48% female with approximately 84% of the population having undergone neutering by their initial study visit. The median enrolment body weight was 20.0 kg (2.5 – 71.5 kg) and the median body condition score was 5/9 (range 3–7). One hundred and twenty eight breeds were represented and 47% of the population were described as mixed breed. The median values for all serum biochemistry and complete blood count parameters were within the applicable reference interval. For certain analytes including serum glucose, amylase, cholesterol, phosphorus, creatine phosphokinase, precision pancreatic lipase, platelet count, haematocrit, and haemoglobin more than 5% of dogs had results outside the reference intervals. On review only 0.25% of dogs were subsequently excluded from continuing the MPB study because the results were considered of clinical significance.

**Conclusions:**

The MPB aims to enable research to deliver insights applicable to the general dog population accessing primary veterinary care in the USA, and recruits accordingly. These data suggest that the first 2,000 dogs recruited in the MPB are comparable in demographics to other studies of the US population. The number of blood test results falling outside of reference intervals (up to 17% depending on analyte), for dogs deemed by veterinarians to be healthy in the context of the clinical history and examination, raises questions around the definition of health and how reference intervals are used. Data gathered during the study is expected to provide valuable information to studies pertaining to genetic, metagenomic, metabolic, dietary, and environmental risk factors associated with early signals of transition to various common diseases.

**Supplementary Information:**

The online version contains supplementary material available at 10.1186/s12917-026-05419-6.

## Background

The Mars Petcare Biobank (MPB) is a population-based prospective cohort study with the aim of analysing longitudinal data from thousands of cats and dogs recruited over a ten year period, to identify novel and actionable insights into pet health. Human biobanking has been progressing for decades, with, for example, the Framingham Heart Study that began in 1948, establishing key risk factors for cardiovascular disease including high blood pressure and high blood cholesterol in the 1960 s [1]. Moving in step with computational data science and biotechnological advances, human biobanks in general have transitioned from being epidemiological studies to also serving as critical tools for biomarker generation and development of precision medicine solutions for complex diseases [2]. For companion animals including dogs, the lack of reliable data and sample sets at a scale relevant to wider populations has been a significant barrier to health and broader welfare research. The veterinary field has lagged significantly in having such access, but there is now a similar but accelerated pattern evident over the last 15–20 years. A few large epidemiological initiatives including VetCompass and SAVSNET in the UK [3, 4], have gathered primary practice records and diagnostic data, and a small number of biobanks including The Golden Retriever Lifetime Study (GRLS) and the Canine Longevity Consortium Dog Aging Project (DAP) have gathered data and some sample sets. While the GRLS is a breed specific study focused on cancer and other common diseases [5–8], the DAP is a large general population study with the aim of identifying the genetic and environmental factors affecting aging, disease prevalence, and development [9–11]. These and other studies have provided evidence for disease risk factors including age, sex, neuter status, breed, and diet [11–15]. The next step, to facilitate prevention and early detection of canine health issues and development of personalised interventions, has several requirements that the MPB has been designed to address.

The range of Mars Petcare businesses, including research and development, diagnostic labs and veterinary hospitals provide a unique opportunity to resource an initiative in which there is the capability and capacity to control sample pipelines and ensure data quality from pets being seen in Mars Veterinary Health (MVH) hospitals. The study aims to enrol a cohort more representative of the wider population that access primary care; collect samples that are handled and stored to standards appropriate for multiomic analyses; gather detailed quality of life, diet, health and environmental information from owners; and reliably link electronic medical records and laboratory data. To maximise this opportunity to deliver insights applicable to as wide a range of dogs as possible, we must assess whether the aim of enrolling a cohort closely resembling the wider population that access veterinary care is being met. This report presents descriptive characteristics of the first 2,000 dogs attending MPB enrolment visits and considers how representative they are of the reported general population in the United States.

## Methods

### Study design

The present study summarises the baseline demographic, haematologic, and serum biochemistry data recorded for the first 2000 dogs enroled in the MPB study through Mars Veterinary Health (MVH) hospitals in the USA between March 2022 and December 2024. The background, design, and powering of the MPB study were previously described in detail [16]. Briefly, informed consent was obtained from the pet owners, and an annual veterinary visit where data was recorded (age, sex, breed, weight, BCS, neuter status, supplements, and annual physical exam) in the study’s electronic data capture system (EDC) as described previously [16]. Blood was collected, routine serum biochemistry, and haematology performed and samples banked annually.

### Setting

Dogs were recruited through MVH hospitals (VCA™ Animal Hospitals, Banfield^®^ Pet Hospitals and BluePearl™ Specialty and Emergency Pet Hospitals). The majority (93%) of the first 2,000 dogs were recruited while attending preventative healthcare appointments at fifty-two primary care hospitals while a minority (7%) were healthy staff pets or blood donors visiting BluePearl™ specialty and emergency hospitals. The dogs were recruited from 21 states across four regions of the United States: South (Arkansas, Florida, Georgia, Kentucky, Louisiana, Maryland, North Carolina, Oklahoma, Tennessee, Texas, and Virginia), West (Arizona, California. Colorado, Hawaii, Oregon and Washington), Midwest (Illinois and Minnesota), and Northeast (New Hampshire and Pennsylvania). Data recording was facilitated by the study’s EDC (Castor EDC, Amsterdam, the Netherlands).

### Eligibility

The aim of the MPB is to enrol otherwise healthy pets and to follow them throughout their lives as some remain healthy. Detailed eligibility criteria are described in additional file.1.0. Briefly, dogs were recruited between the ages of 6 months and 10 years. Male and female neutered or entire dogs with a body weight ≥ 2.5 kg (to ensure the required blood volume is well within 5% of circulating volume) were recruited if their body condition score (BCS) was 3–7 on a 9-point scale as determined by the attending veterinary professional [17]. All study pets were otherwise healthy at enrolment without diagnosed or suspected, acute or chronic medical conditions. The attending veterinarian reviewed the enrolment blood results alongside the clinical examination to confirm that the animal met the eligibility criteria. Those where pathology was suspected after review of the blood work were withdrawn from the MPB study.

### Data collection

The demographic data and fasting status were recorded for each dog by the veterinary professional during the enrolment visit. Pet age was calculated either from the known date of birth, or the known or estimated year of birth (known *n* = 1600 or estimated *n* = 400). Where only year of birth was available, the date of birth was set as January 1 st of that year. If neuter status was unknown, the dog was classified as intact. Breed was recorded by the attending veterinary professional at the enrolment visit by selecting from a standardised breed list; those assigned a single breed were classified as purebred with no requirement for Kennel Club registration. Any dog reported to be a mix or cross was classified as mixed breed. The results of serum biochemistry and complete blood counts were from samples collected at the enrolment visit. Standard veterinary care was provided at each sample collection but if blood samples were not successfully collected or were not suitable for analysis due to quality or volume, the protocol asked for a repeat blood draw to be made within three weeks of the veterinary exam. Blood results received more than three weeks after the initial appointment were excluded from the analyses as demographic and clinical data may have changed.

Routine blood tests were performed at Antech® Diagnostics reference laboratories local to the hospitals. Serum biochemistry included precision pancreas-specific lipase (pPSL), total protein (T. Protein), albumin, globulin, total bilirubin (T. Bilirubin), gamma-glutamyl transferase (GGT), alkaline phosphatase (ALP), alanine aminotransferase (ALT), aspartate aminotransferase (AST), creatine phosphokinase (CK), phosphorus, blood urea nitrogen (BUN), creatinine, BUN/creatinine ratio, glucose, calcium, magnesium, sodium, potassium, chloride, cholesterol, triglycerides, and amylase. The semiquantitative serum lipemic, icteric and hemolytic index (LIH) were also reported, although these factors can affect the measurement of some analytes they were not used to exclude data from statistical analyses. The complete blood count measured absolute basophils, eosinophils, lymphocytes, monocytes, neutrophils, white blood cells (WBC), red blood cells (RBC), haemoglobin (HGB), haematocrit (HCT), mean corpuscular volume (MCV), mean corpuscular haemoglobin concentration (MCHC), mean corpuscular haemoglobin (MCH), and platelet count.

### Statistical analysis

Descriptive statistics including median, range and interquartile ranges were calculated for continuous/ordinal variables such as age, body weight and body condition score, while proportions were reported for categorical variables including breed, sex, neuter status, and geographical region. For routine serum biochemistry and complete blood count, minimum, and maximum values, mean, median and standard deviation were calculated. Upon review of the initial analytic results, we focused on parameters that have clinical importance, and compared to other measured values, exhibited a notably higher proportion of results falling outside the established reference interval. Cholesterol and phosphorus were selected to undergo further inferential statistical analysis. The effect of fasting state (recorded in the EDC system at the visit as fasted, not fasted, or unknown) and breed on serum cholesterol were explored using one-way ANOVA tests. The correlation between BCS and serum cholesterol was explored using Spearman’s ranked correlation test. The correlation of age and serum phosphorus level was tested by Pearson's correlation. All analyses were performed using R Statistical Software (v4.2.2; R Core Team 2021).

## Results

**Fig. 1:**
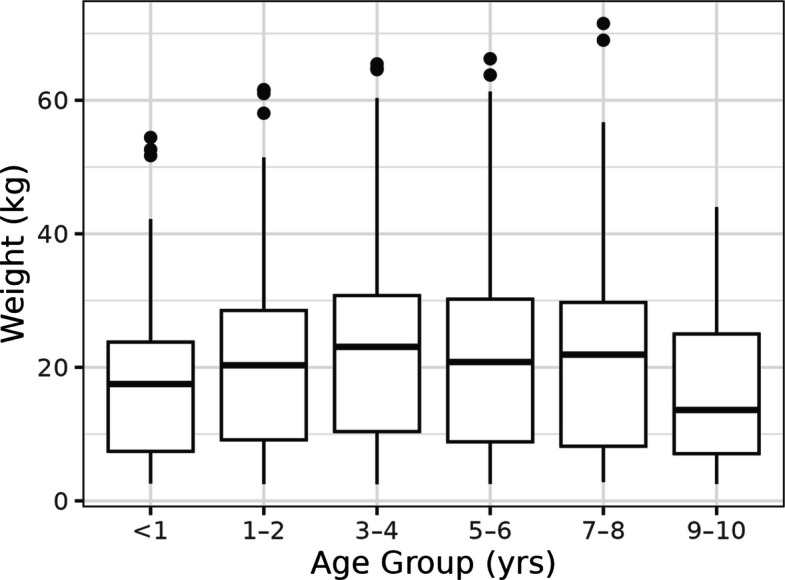
Body weight distribution by age

**Fig. 2:**
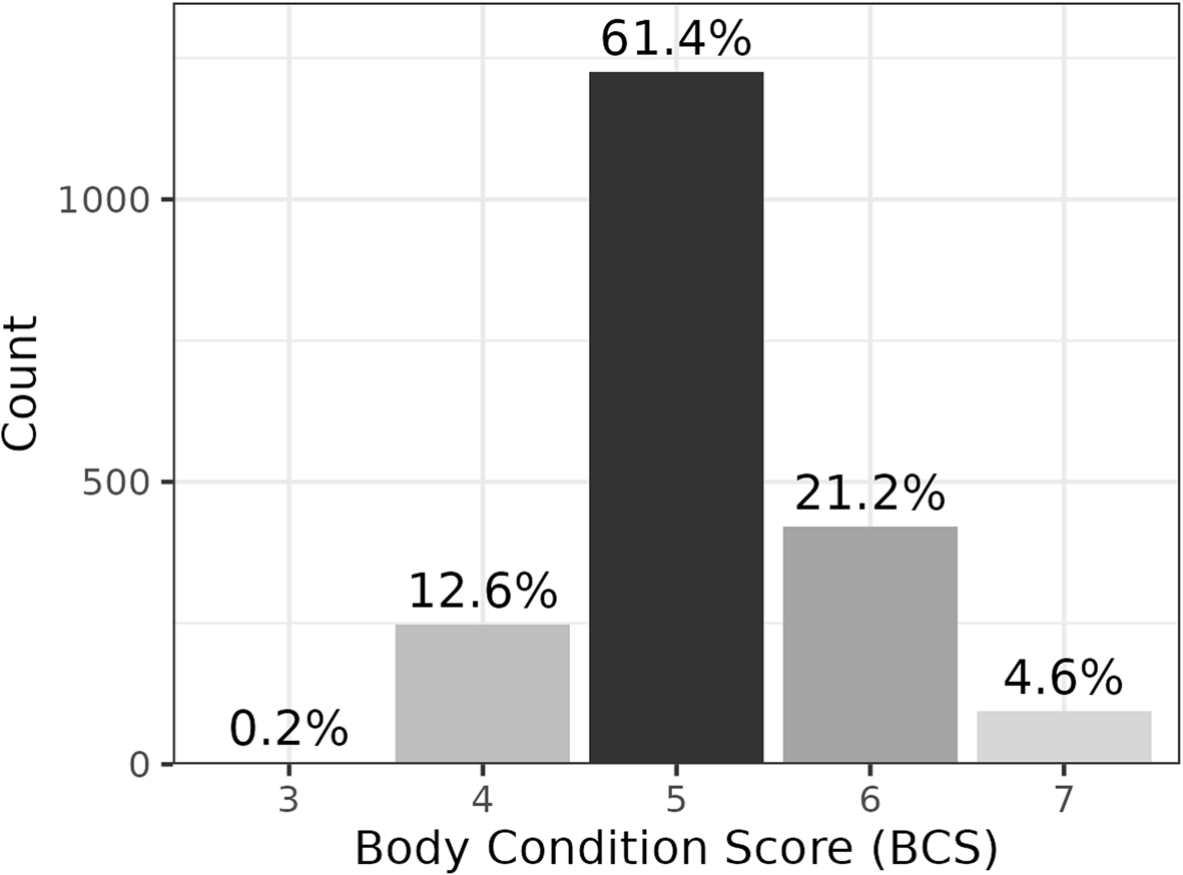
Body condition score distribution. BCS: body condition score, values above the bars indicate % of population with that score, *n* = 1991 dogs

One hundred and twenty-eight breeds were reported in the population, with 47.3% of dogs described as mixed breed (Table 1). The median enrolment age of the first 2,000 dogs was 3.0 yrs. (0.5–10.0 yrs.) with 25% of the population between 0.5 and 1.5 yrs. and 25% between 5.2 and 10.0 yrs (Additional file 2.0). The median enrolment body weight was 19.8 kg (2.5–71.5); when stratified by age, dogs aged between 9 and 10 years had the lowest median body weight of 11.3 kg (2.5–40.8) and those aged 3–4 years had the highest of 22.8 kg (2.5–65.5) (Fig. 1, Table 2). The median BCS for the population was 5/9 (range 3—7) with 61.4% given this score and 25.8% given a BCS of 6 or 7 (Fig. 2). The population was evenly distributed for sex with 47.9% female and 52.1% male dogs, 85.4% of the population had undergone neutering before the enrolment visit, with the highest percentage of intact dogs (42.2% of males and 25.4% of females) being in the youngest age group (Table 2). Location, stratified by geographical region of the USA, was based on the address of the recruiting hospital and 0.4% of first 2000 participant dogs were recruited from the Midwest, 2.3% from the Northeast, 65.9% from the South and 31.4% from the West (Additional file 3.0).

**Table 1:** Number and percentage of mixed, purebred and the top 10 breeds in the first 2,000 dogs enroled in the Mars Petcare Biobank

Reported Breed	Number	Percentage of population
Mixed or cross breed	945	47.3
Pure bred	1055	52.8
Labrador Retriever	93	4.7
Golden Retriever	76	3.8
Poodle (all sizes)	62	3.1
Australian Shepherd	50	2.5
Shih Tzu	49	2.5
American Staffordshire Terrier	48	2.4
Chihuahua	44	2.2
Yorkshire Terrier	36	1.8
French Bulldog	32	1.6
German Shepherd Dog	31	1.6

**Table 2:** Body weight, sex, and neuter status distribution by age

Age group		Male	Female	Male Neutered	Female Spayed	Median body weight
(yrs.)	*n*	n (%)	n (%)	n (% of males)	n (% of females)	kg (range)
All	2000	1042.0 (52.1%)	958.0 (47.9%)	852.0 (81.8%)	855.0 (89.2%)	19.8 (2.5–71.5)
< 1	261	135.0 (51.7%)	126.0 (48.3%)	78.0 (57.8%)	94.0 (74.6%)	16.0 (2.6–54.4)
1–2	704	367.0 (52.1%)	337.0 (47.9%)	295.0 (80.4%)	293.0 (86.9%)	20.0 (2.5–64.0)
3–4	448	234.0 (52.2%)	214.0 (47.8%)	203.0 (86.8%)	198.0 (92.5%)	22.8 (2.5–65.5)
5–6	355	182.0 (51.3%)	173.0 (48.7%)	162.0 (89.0%)	168.0 (97.1%)	20.3 (2.5–63.8)
7–8	192	100.0 (52.1%)	92.0 (47.9%)	92.0 (92.0%)	86.0 (93.4%)	21.4 (2.8–71.5)
9–10	40	24.0 (60.0%)	16.0 (40.0%)	22.0 (91.7%)	16.0 (100%)	11.3 (2.5–40.8)

Dogs stratified by age *n* = 2,000

Serum biochemistry results were available for 1,766 dogs, and CBC results for 1,864. The remainder were either from dogs where a sample was not successfully collected within the required timeframe or quality was inadequate. After review of blood and clinical examination results by the attending veterinarian, five dogs were withdrawn from continuing the MPB study. No serum samples were recorded as icteric, 0.8% had a lipemic index of 3 to 5 (200 — > 500 mg/dL lipid), and 7% had a haemolytic index of 3 to 5 (200 — > 500 mg/dL haemoglobin) (Additional file 4.0). Median values for all serum biochemistry and complete blood count parameters were within the reference intervals reported by Antech Diagnostics with no adjustments made for age or breed (Table 3). For some parameters including serum glucose, amylase, cholesterol, phosphorus, CPK, pPSL, platelet count, HCT and HGB more than 5% of dogs had results outside the reference interval (Table 3). Parameters that have clinical importance and compared to other measured values exhibited a notably higher proportion of results falling outside the established reference interval were selected to undergo further inferential statistical analysis. Fasted state was found to have a significant effect on serum cholesterol level (ANOVA: F = 3.4; d.f. = 2; *n* = 1922, *p* = 0.03) as did breed (ANOVA: F = 4.6; d.f. = 19; *n* = 1011 *p* < 0.001). A weak but statistically significant positive correlation between BCS and serum cholesterol (Spearman’s rho = 0.13, *n* = 2000, *p* < 0.001) was determined and a negative correlation between age and serum phosphorus level (*r* = −0.44, *n* = 1794, *p* < 0.001) was also observed.

**Table 3:** Routine serum biochemistry and complete blood count values with reference intervals

Measure	N	Mean	Median	SD	Min	Max	Reference interval (RI)	% within RI	% below RI	% above RI
Albumin (g/dl)	1,766	3.6	3.6	0.3	2.5	7.9	2.7–4.4	98.6	0.3	1.1
ALP (U/l)	1,763	49.3	34.0	65.4	5.0	1,212.0	5.0–131.0	95.6	0.0	4.4
ALT (U/l)	1,764	42.5	35.0	36.2	4.0	777.0	12.0–118.0	97.6	0.2	2.2
Amylase (U/l)	1,764	490.4	458.5	166.4	153.0	1,637.0	290.0–1,125.0	93.8	5.5	0.7
AST (U/l)	1,765	29.8	27.0	20.6	9.0	622.0	15.0–66.0	98.1	0.9	1.1
BUN (mg/dl)	1,765	17.6	17.0	5.0	6.0	51.0	6.0–31.0	98.5	0.0	1.5
BUN/Creatinine	1,765	18.3	17.0	6.9	5.0	90.0	4.0–27.0	91.5	0	8.6
Calcium (mg/dl)	1,766	10.2	10.2	0.6	4.3	12.6	8.9–11.4	95.8	2.9	1.3
Chloride (mEq/dl)	1,765	113.0	113.0	3.0	82.0	129.0	102.0–120.0	99.3	0.1	0.6
Cholesterol (mg/dl)	1,765	242.2	237.0	64.1	98.0	1,100.0	92.0–324.0	90.1	0.0	9.9
CPK (U/l)	1,764	178.0	120.0	526.8	24.0	18,519.0	59.0–895.0	93.1	6.1	0.8
Creatinine (mg/dl)	1,766	1.0	1.0	0.3	0.3	2.3	0.5–1.6	98.2	0.7	1.1
GGT (U/l)	1,764	5.0	4.0	2.8	1.0	27.0	1.0–12.0	99.7	0	0.3
Globulin (g/dl)	1,766	2.7	2.6	0.5	1.5	7.4	1.6–3.6	96.9	0.2	2.9
Glucose (mg/dl)	1,766	88.3	93.0	21.9	10.0	186.0	70.0–138.0	86.2	13.7	0.2
Magnesium (mEq/dl)	1,766	1.9	1.9	0.3	1.3	8.5	1.5–2.5	98.1	0.8	1.1
NA/K	1,765	32.7	33.0	2.8	14.0	44.0	27.0–38.0	96.5	1.5	2.0
Phosphorus (mg/dl)	1,765	4.4	4.2	1.4	1.2	17.6	2.5–6.0	86.1	2.8	11.1
Potassium (mEq/dl)	1,765	4.6	4.5	0.4	3.5	9.5	3.6–5.5	98.3	0.2	1.5
Precision PSL (U/l)	1,766	62.6	46.0	59.7	9.0	755.0	24.0–140.0	82.9	10.0	7.1
Sodium (mEq/dl)	1,766	148.5	149.0	2.8	126.0	165.0	139.0–154.0	98.4	0.5	1.2
T. Bilirubin (mg/dl)	1,766	0.2	0.1	0.6	0.1	24.7	0.1–0.3	97.2	0.0	2.8
T. Protein (g/dl)	1,766	6.3	6.3	0.6	4.4	15.3	5.0–7.4	97.4	0.4	2.2
Triglyceride (mg/dl)	1,765	94.6	65.0	125.8	13.0	2,474.0	29.0–291.0	95.6	1.2	3.2
Abs. Basophils (/ml)	1,864	0.9	0.0	15.0	0.0	326.0	0.0–150.0	99.7	0.0	0.3
Abs. Eosinophils (/ml)	1,864	448.8	360.0	413.8	0.0	5,952.0	0.0–1,200.0	96.1	0.0	3.9
Abs. Lymphocytes (/ml)	1,864	2,402.8	2,184.0	1,052.2	320.0	7,912.0	690.0–4,500.0	94.9	0.5	4.7
Abs. Monocytes (/ml)	1,864	407.9	360.0	214.8	0.0	2,706.0	0.0–840.0	95.9	0.0	4.1
Abs. Neutrophils (/ml)	1,864	5,586.7	5,220.0	1,984.4	1,581.0	20,418.0	2,060–10,600.0	97.8	0.4	1.8
HCT (%)	1,864	53.2	54.0	5.6	29.0	73.0	36.0–60.0	91.4	0.3	8.3
HGB (g/dl)	1,864	17.7	17.8	1.9	10.0	24.3	12.1–20.3	92.3	0.6	7.1
MCH (pg)	1,864	24.6	24.6	1.2	14.5	30.6	19.0–28.0	99.7	0.1	2.1
MCHC (g/dl)	1,864	33.3	33.0	1.2	28.0	38.0	30.0–38.0	99.5	0.5	0.0
MCV (fl)	1,864	74.0	74.0	3.6	44.0	90.0	58.0–79.0	94.6	0.0	5.4
Platelet Count (10^3^/ml)	1,864	256.5	249.0	86.1	26.0	835.0	170.0–400.0	84.0	11.1	5.0
RBC (10^5^/ml)	1,864	7.2	7.2	0.8	4.0	9.8	4.8–9.3	99.5	0.2	0.3
WBC (10^3^/ml)	1,864	8.8	8.5	2.6	3.2	24.6	8.8–15.5	97.6	0.5	1.9

*SD* Standard deviation, *ALP* Alkaline phosphatase, *ALT* Alanine aminotransferase, *AST* Aspartate aminotransferase, *BUN* Blood urea nitrogen, *CPK* Creatine phosphokinase, *GGT* Gamma-glutamyl transferase, *Na/K* Sodium/potassium ratio, *pPSL* precision pancreatic lipase, *T. bilirubin* Total bilirubin, *T. protein* Total protein. *Abs* Absolute, *HCT* Haematocrit, *HGB* Haemoglobin, *MCHC* mean corpuscular haemoglobin concentration, *MCH* mean corpuscular haemoglobin, *MCV* mean corpuscular volume, *RBC* Red blood cells, *WBC*: White blood cells

Box and whisker plot showing body weight grouped by age, *n* = 2000. Horizontal lines represent the median, boxes represent the 25th to 75th percentile and whiskers represent the highest and lowest values that are not outliers. Outliers are represented by circles.

## Discussion

To facilitate research into the prevention and early detection of canine health issues and the development of personalised interventions, the MPB has been designed to enrol a cohort closely resembling the general healthy population that accesses veterinary care. Results of this first analysis indicate that to date, our cohort is demographically representative. In the first 2,000 dogs, the ten most frequent breeds aligned with those reported as most popular by The American Kennel Club [18], and trends were similar to those reported in other recent studies [11, 19, 20]. Half of the MPB population being described as mixed breed is also comparable to the reported make up (52% purebred and 48% mixed breed) of the DAP cohort of 21,410 US dogs [21].

Breed preferences, as well as disease prevalences, may be associated with geographical location. Many breeds exhibit a regionally constrained genetic pool [22], and it has been suggested that geographic differences in breed specific mortality may exist [23]. To meet the aims of the study, participants should therefore be representative of the geographic location of the general population. However, the geographic location of the first 2,000 enrolees was skewed towards the South of the USA with approximately one third recruited in Florida. This reflects the locations of the first recruiting hospitals as well as those initially most successful in enrolling participants. As recruitment progresses, the population distribution will be compared to published figures for household pet ownership by geographical location e.g. Pet Ownership Statistics by State 2025 [24], and numbers of dogs attending MVH hospitals. Interventions to spread recruitment appropriately, for example by the introduction of new hospital locations, will be made.

The balanced proportion of female (48%) to male (52%) dogs also agreed with previous cohorts including 20,970 dogs attending three private hospitals [19] and 13,292,929 dogs attending over 1,000 Banfield Pet Hospitals in the United States [25]. The highest percentages of intact male and female dogs (42.2% and 25.4% respectively) were observed in those aged less than 1 year old which is unsurprising given that clinical guidelines suggest that sterilisation should be recommended between 5 and 15 months of age depending on sex and breed size [26]. The number of intact dogs in our study could be overestimated because those of unknown neuter status were regarded as intact. However, the percentage of dogs neutered before enrolment (~ 85%) was comparable to the 80–95% documented in other recent studies [19, 21]. A lower percentage (64%) of neutered dogs was reported in a 2007 study of 1,339,860 dogs examined at 651 veterinary hospitals in the United States [27]. Attitudes to sterilisation may have changed since this study [28] and it was noted that dogs with preventative health care plans were more likely to be neutered, which applies to most pets in the MPB enroled though Banfield and VCA hospitals.

As a healthy status is required, the low median age of the MPB population at enrolment (~ 3 yrs.) with a quarter between the ages of 6 months and 1.5 years, was not unexpected. A further quarter of the initial 2,000 recruited pets aged between 5.2–10.0 yrs provide an early opportunity to study factors affecting healthy ageing.

The median body weight of the first 2,000 enrolees was 20.0 kg, similar to that reported by the DAP, a larger cohort of pure and mixed breed dogs in the United States, for both pure (24.0 kg) and mixed breed subjects (21.6 kg) [20]. As expected, the median body weight of dogs less than one year of age was lower than that of older dogs up to nine years. This is likely due to some not having reached their adult body weight at the time of enrolment as this may not be achieved until 15 months of age for large breeds [29]. Furthermore, older dogs are more likely to be overweight as excess body weight becomes particularly prevalent in middle aged dogs [30, 31]. However, the lowest median body weight was that of dogs aged between nine and ten years and may be attributed to the longer lifespan and health span of smaller dogs [11, 32] confirming that they are more likely to be healthy and eligible for enrolment when older. In addition, body weight varies among breeds, although the dogs in this cohort were mostly larger mixed or pure breeds, stratification to investigate the effect of breed on body weight will be included in future studies to provide insights.

In a study of over 1.9 million adult dogs visiting Banfield Pet Hospitals, 51% were classified as overweight [33]. However, to encourage recruitment, the MPB study determined that only dogs with a BCS ranging from 3–7/9 were eligible for recruitment. Although this approach might introduce certain bias and limitations, the authors deemed that this approach did not undermine the value of the data presented here as the epidemiology of obesity was out of the scope at this time. Dogs moderately above ideal BCS were enroled if the attending veterinarian determined no evidence of associated disease and was confident that the dog was healthy. These criteria do exclude dogs with a BCS of 8–9/9, which may represent a significant proportion of the general canine population. However, as these dogs are classified as obese, they do not meet the criteria for inclusion in an otherwise healthy population. The median enrolment body condition score of the population was 5 with ~ 75% of dogs scoring four or 5 i.e. an “ideal” BCS. However, a quarter of the first 2,000 dogs had a BCS of 6 or 7, with each additional score representing a 5% increase in body fat [17, 34]. As part of the future longitudinal follow-up, all enroled dogs will have their BCS monitored at each annual visit. Any changes, including the development of obesity, will be documented and analysed in future reports, allowing interpretation of health outcomes in the context of evolving BCS.

As illustrated by lab work results outside the reference intervals and the decision of attending veterinarians regarding eligibility at recruitment, precisely determining whether an individual is healthy is not always simple and straightforward. In human medicine the definition of ‘health’ has been a matter of significant debate. The World Health Organisation constitution defines health as ‘a state of complete physical, mental and social wellbeing and not merely the absence of disease or infirmity’ [35]; although much of this is difficult (although not impossible) to measure for animals. One analysis of 500 veterinary textbooks found that most did not contain an explicit definition of health and/or disease, and there is an often erroneous view of these as distinct, opposite states (the ‘health and disease binary’) [36]. In human medicine it has been observed that the number of laboratory results outside the “normal range” far exceeds the clinically meaningful abnormal results due to the usually accepted methodology for ascertaining “normal values”/reference ranges, variations in methods of testing at different laboratories, variations due to age, gender, ethnicity, seasonality, and random variations [37]. Here and in previous studies this creates complexity in defining health, particularly when using diagnostic test panels. In the current analysis, all median/mean values for serum biochemistry and complete blood count data were within the reference intervals for the analysis methodologies used. For some parameters more than 5% of values were outside of the reference interval. Similar results were also reported in a study of 196 young adult dogs (median age 2 years) defined as being healthy for three or more years in the GRLS, where 0.8–7.7% of values fell outside various reference intervals [38]. There are well-known pre-analytical causes for such variability including, fasting state, phlebotomy technique, underfilling of EDTA tubes, haemolysis or lipemia [39–42]. Approximately 12% of serum biochemistry and 7% of complete blood count data were regarded as missing at random because they were not provided within three weeks of the enrolment visit (a time restriction put in place to allow linkage of metadata such as body weight and BCS to the blood results). Reasons for this included blood sampling not being possible for behavioural reasons, failed phlebotomy, insufficient volume collected, sample quality issues, and shipping delays; factors also reported to have increased levels of missing data in other studies [38, 43]. In these cases, a repeat blood sample was requested, however, due to limited availability of follow up veterinary technician appointments and the willingness of owners to attend a second visit, a percentage of data was collected outside of the time limits. Time delays can also influence results, for example artefactual hypoglycaemia due to continued glucose consumption via blood cell glycolysis [40, 44] and platelet aggregation [40, 41]. The MPB study protocol is designed to minimize the impact of these factors as much as possible [16]. However, because samples are collected in a real-world clinical setting rather than a controlled research environment, preanalytical variation is more challenging to manage. Consequently, the protocol will be periodically reevaluated to address issues related to missing samples and to identify potential causes of sample delays. There are also physiological/demographic reasons for analyte levels falling outside of reference intervals. As reported previously [45], increased serum cholesterol was associated with higher BCS (subcohort noted above). Breed also influenced serum cholesterol as some breeds, including Golden Retrievers and Miniature Schnauzers, have naturally higher circulating lipid levels [46, 47]. Higher serum phosphorus levels were noted in younger dogs (a large proportion of this cohort), [40, 46], reflecting the effect of increased concentrations of growth hormone on bone turnover and renal phosphate reabsorption up to two to four years of age [48]. It has also been shown that clinically meaningful changes in analytes e.g. associated with ageing can occur within reference intervals [49]. Because of the limitations in the application of reference intervals it has been suggested that clinical interpretation could be assisted by the application of subject-based reference values that define the variability in a healthy individual owing to analytical and biological variation [38, 50, 51]. Just as health and disease are not dichotomous, population-based reference intervals may not be arbiters of whether disease is present or not [51]. For that reason, the decision on whether a dog is considered to be healthy and therefore eligible for enrolment in the MPB is made by the examining veterinarian, who makes that decision within a broader clinical context.

### Limitations

The aim of the study is to recruit a cohort closely resembling the general healthy dog population attending veterinary hospitals in the United States. Our ability to assess progress against this aim is however constrained by the lack of national demographic databases and limited published up-to-date and large-scale empirical data regarding domestic dog demographics in the USA.

For a quarter of the cohort, the exact date on birth was not known and so was estimated leading to potential inaccuracy. In the future, age may be better estimated using epigenetic techniques reported previously to have a median error of less than seven months for chronological age [52]. A proportion of dogs were reported as above or below their ideal BCS, and for these body weight may not be used as a direct surrogate for breed size. Although standard BCS charts were made available to veterinary staff at the time of examination via the study EDC system, no attempt was made to cross validate scores within or between scorers and discordances between body condition score and clinical diagnosis have been noted in previous studies [31]. It is therefore likely that some intra and inter-operator variability in scoring is present particularly across breeds of different morphologies [53]. Breed was recorded by the veterinary professional selecting from a standardised list, however visual identification of dog breed by owners or veterinary professionals has been reported to be potentially inaccurate [54]. This has the potential to be determined more accurately using genotyping data derived from Wisdom Panel™ canine DNA breed testing (Wisdom Health, Vancouver, WA, USA).

Finally, a common limitation of cohort studies, which also applies to the MPB, is that owners enrolling their dogs are those that are motivated and able to regularly engage with veterinary care and therefore not necessarily representative of the general pet owning population. There may also be differences in patient populations between Banfield Pet Hospitals, VCA Animal Hospitals, and BluePearl Specialty and Emergency Pet Hospitals. In the future, when the dataset contains sufficient sample size within specific patient groups, stratification by hospital will be possible to explore this. In human biobanks such as the UK Biobank statistical methods to correct for selection biases by inverse probability weighting have recently been developed [55] and this may be applied in subsequent research to mitigate selection bias.

## Conclusion

The MPB is recruiting a healthy cohort that is currently considered to be representative of the United States healthy dog population as reported in terms of age, size, sex, neuter status, breed, and clinical pathology. Increased regional coverage will be prioritised going forward to ensure that the study will provide representative new data to elucidate risk factors for later disease, early disease markers, personalised medicine approaches, and accelerate studies into comorbidities, factors affecting healthy aging, and treatment outcomes. In this way the MPB will provide a unique opportunity to advance veterinary healthcare.

## Supplementary Information

Supplementary Material 1.

Supplementary Material 2.

Supplementary Material 3.

Supplementary Material 4.

## Data Availability

The datasets generated and/or analysed during the current study are not publicly available as they are proprietary but are available from the corresponding author on reasonable request. Researchers wishing to initiate research based on the MPB are invited to contact the Biobank (info@marspetcarebiobank.com) to obtain access for specific research proposals.

## Abbreviations

ANOVA: Analysis of variance
BCS: Body condition score
DAP: Dog Aging Project
d.f.: Degrees of freedom
EDC: Electronic data capture system
EMR: Electronic medical record
GRLS: Golden Retriever Lifetime Study
MPB: MARS PETCARE BIOBANK
MVH: Mars Veterinary Health
SD: Standard deviation
ALP: Alkaline phosphatase
ALT: Alanine aminotransferase
AST: Aspartate aminotransferase
BUN: Blood urea nitrogen
CPK: Creatine phosphokinase
GGT: Gamma-glutamyl transferase
Na/K: Sodium/potassium ratio
pPSL: Precision pancreas-specific lipase
T. bilirubin: Total bilirubin
T. protein: Total protein
Abs: Absolute
HCT: Haematocrit
HGB: Haemoglobin
MCHC: Mean corpuscular haemoglobin concentration
MCH: Mean corpuscular haemoglobin
MCV: Mean corpuscular volume
RBC: Red blood cells
WBC: White blood cells
