# Supplementary material for: Cohort profile of the first 2,000 canine enrolees in the Mars Petcare Biobank: demographic, hematologic and serum biochemistry results from March 2022 to December 2024
Source: BMC Vet Res. 2026 Mar 20;22:252. doi: 10.1186/s12917-026-05419-6 (PMC13123173; doi:10.1186/s12917-026-05419-6)
Supplement: Supplementary file 1 — Supplementary Material 1. [file 12917_2026_5419_MOESM1_ESM.docx]

### Additional file 1.0.

### Enrolment eligibility criteria

Inclusion:

- Canine, male or female, any neuter status.
- Age greater than or equal to 6 months and less than or equal to 10 years.
- Current body weight greater than 2.5 kgs.
- Body condition score between 3 to 7 on a 9-point scale.
- Apparent good health as determined by the primary veterinarian based on comprehensive physical examination and owner discussion.
- Brachycephalic dogs assessed as healthy through comprehensive clinical evaluation.
- Predisposition to genetic disease if no clinical signs or relevant medical history of the condition.
- Infrequent use of anti-anxiety medications (e.g., voluntary pre-appointment medications)
- Receiving regular prophylactic flea, tick, and deworming (including heartworm) treatments and/or over-the-counter medications, including those reported to elicit calming effects.
- Mild/moderate (up to stage 2) periodontal disease: such as tartar or previous dental extraction.

Exclusion

- Ongoing medical condition deemed by the primary veterinarian to significantly interfere with patient’s quality of life or safety.
- Chronic or concurrent conditions and/or suspected conditions i.e. hypothyroidism, hyperthyroidism, kidney disease, hepatopathy, chronic pancreatitis, diabetes, Cushing’s/hyperadrenocorticism, Addison’s/hypoadrenocorticism, other metabolic disorders, exocrine pancreatic insufficiency, autoimmune diseases and cardiac disease.
- Current and/or historical diagnosis of malignant neoplasia, regardless of successful treatment status.
- Currently receiving prescription medication for treatment of any chronic and/or concurrent medical condition.
- Congenital, hereditary, or genetic conditions, or a history of such conditions, which necessitate ongoing medical management.
- Brachycephalic dogs requiring ongoing medical intervention due to their physical characteristics or genetic predisposition.
- Minor ailments: including seasonal allergies, infectious diseases, parasites, and/or minor injuries currently receiving treatment medication (may be rescreened 60 days after treatment discontinuation).
- Pregnant or less than 12 weeks postpartum.
