# Supplementary material for: Cohort profile of the first 2,000 canine enrolees in the Mars Petcare Biobank: demographic, hematologic and serum biochemistry results from March 2022 to December 2024
Source: BMC Vet Res. 2026 Mar 20;22:252. doi: 10.1186/s12917-026-05419-6 (PMC13123173; doi:10.1186/s12917-026-05419-6)
Supplement: Supplementary file 2 — Supplementary Material 2. [file 12917_2026_5419_MOESM2_ESM.pdf]

Additional file 2.0. Violin plot of age distribution.

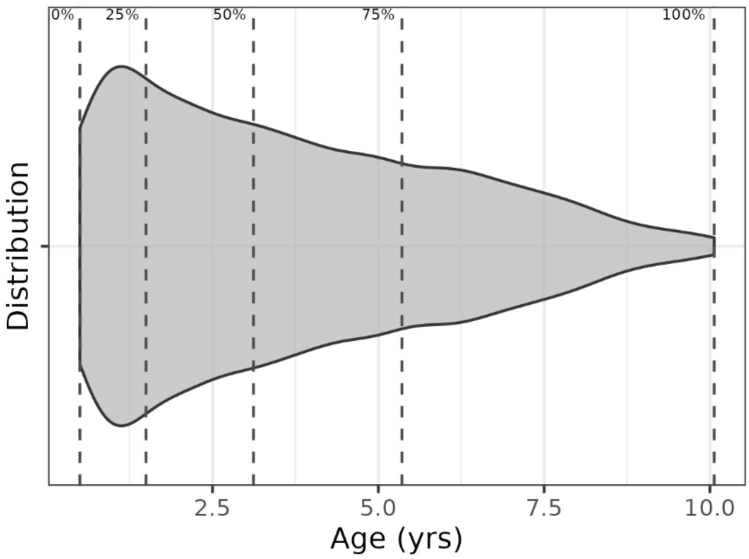

X-axis represents age, y-axis shows the corresponding data distribution i.e. the width of the shaded area represents the proportion of individuals at that age**.** Vertical lines indicating the 25th, 50th, 75th, and 100th percentiles.
