# Supplementary material for: Cohort profile of the first 2,000 canine enrolees in the Mars Petcare Biobank: demographic, hematologic and serum biochemistry results from March 2022 to December 2024
Source: BMC Vet Res. 2026 Mar 20;22:252. doi: 10.1186/s12917-026-05419-6 (PMC13123173; doi:10.1186/s12917-026-05419-6)
Supplement: Supplementary file 3 — Supplementary Material 3. [file 12917_2026_5419_MOESM3_ESM.docx]

Additional file 3.0. Numbers of dogs recruited by US State.


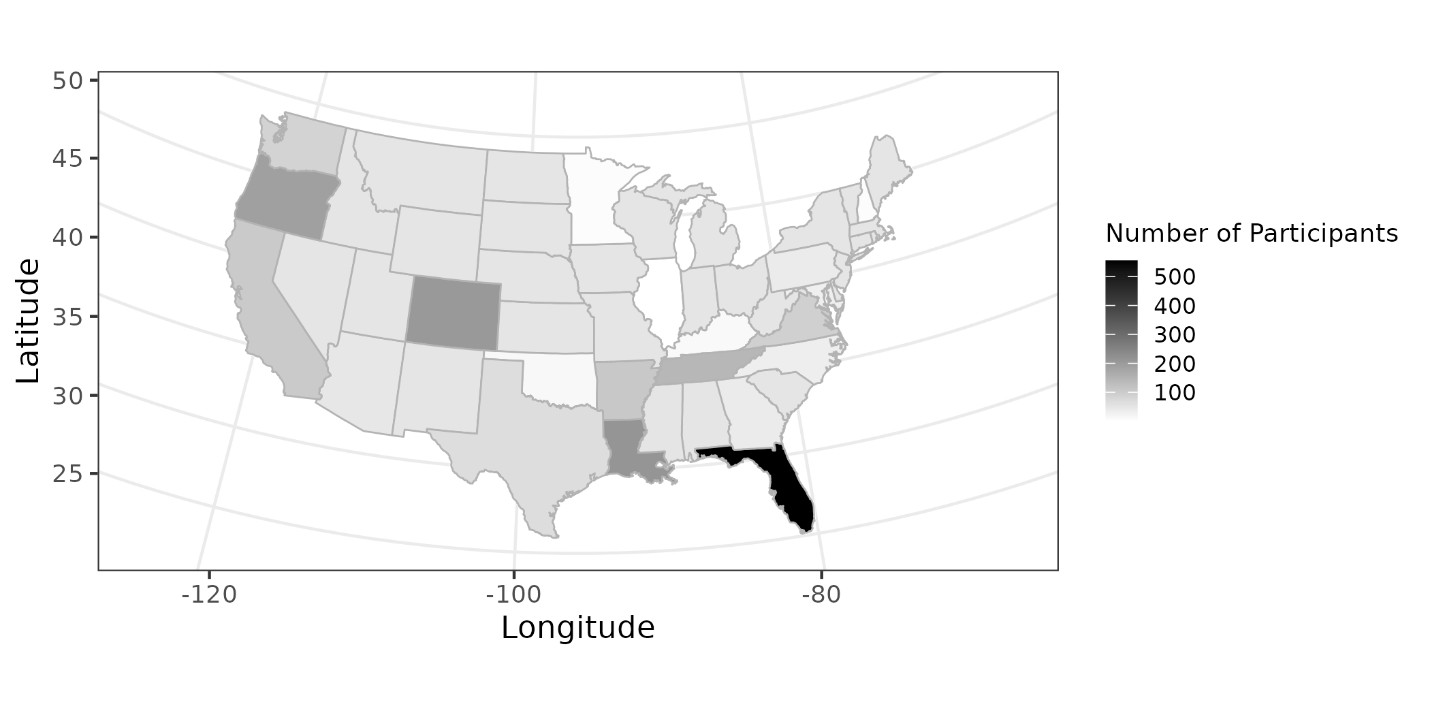


Map of MPB recruitment by geographical region coloured by number of dogs recruited. Includes full population (n= 2000). This map was generated using R Statistical Software (v4.2.2; R Core Team 2021) and ggplot2 version 3.5.1.
