# Supplementary material for: Cohort profile of the first 2,000 canine enrolees in the Mars Petcare Biobank: demographic, hematologic and serum biochemistry results from March 2022 to December 2024
Source: BMC Vet Res. 2026 Mar 20;22:252. doi: 10.1186/s12917-026-05419-6 (PMC13123173; doi:10.1186/s12917-026-05419-6)
Supplement: Supplementary file 4 — Supplementary Material 4. [file 12917_2026_5419_MOESM4_ESM.docx]

Additional file 4.0. Number of serum biochemistry results reporting lipemia, icterus or haemolysis (LIH).

| Number of results at each level | | | | | | |
| --- | --- | --- | --- | --- | --- | --- |
| LIH Index | 0 | 1 | 2 | 3 | 4 | 5 |
| Haemolysis | 848 | 577 | 216 | 59 | 47 | 18 |
| Lipemia | 1,567 | 148 | 36 | 5 | 6 | 3 |
| Icterus | 1,765 | 0 | 0 | 0 | 0 | 0 |

Includes full population (n= 2000)
